# LC-ESI-MS/MS Analysis of Sulfolipids and Galactolipids in Green and Red Lettuce (*Lactuca sativa* L.) as Influenced by Sulfur Nutrition

**DOI:** 10.3390/ijms24043728

**Published:** 2023-02-13

**Authors:** Tania T. Körber, Tobias Sitz, Muna A. Abdalla, Karl H. Mühling, Sascha Rohn

**Affiliations:** 1 Institute of Food Chemistry, Hamburg School of Food Science, University of Hamburg, Grindelallee 117, 20146 Hamburg, Germany; 2 Institute of Plant Nutrition and Soil Science, Kiel University, Hermann-Rodewald-Str. 2, 24118 Kiel, Germany; 3 Institute of Food Technology and Food Chemistry, Technische Universität Berlin, Gustav-Meyer-Allee 25, 13355 Berlin, Germany

**Keywords:** glycolipids, fatty acids, oxidized SQDG, S-limiting condition

## Abstract

Sulfur (S) deprivation leads to abiotic stress in plants. This can have a significant impact on membrane lipids, illustrated by a change in either the lipid class and/or the fatty acid distribution. Three different levels of S (deprivation, adequate, and excess) in the form of potassium sulfate were used to identify individual thylakoid membrane lipids, which might act as markers in S nutrition (especially under stress conditions). The thylakoid membrane consists of the three glycolipid classes: monogalactosyl- (MGDG), digalactosyl- (DGDG), and sulfoquinovosyl diacylglycerols (SQDG). All of them have two fatty acids linked, differing in chain length and degree of saturation. LC-ESI-MS/MS served as a powerful method to identify trends in the change in individual lipids and to understand strategies of the plant responding to stress. Being a good model plant, but also one of the most important fresh-cut vegetables in the world, lettuce (*Lactuca sativa* L.) has already been shown to respond significantly to different states of sulfur supply. The results showed a transformation of the glycolipids in lettuce plants and trends towards a higher degree of saturation of the lipids and an increased level of oxidized SQDG under S-limiting conditions. Changes in individual MGDG, DGDG, and oxidized SQDG were associated to S-related stress for the first time. Promisingly, oxidized SQDG might even serve as markers for further abiotic stress factors.

## 1. Introduction

Lettuce is an important fresh-cut vegetable that, in addition to its cultivation on the field, can be grown in hydroponic systems. Such systems are becoming increasingly important and have the advantage of optimizing plant nutrition specifically for particular plants. Consequently, yield, dry matter (DM), and various health-related parameters can be increased. Thus, it is possible to improve food quality during the cultivation of plants [1]. The use of hydroponic systems allows a targeted adjustment of macro- as well micronutrients. In addition, other growth conditions (e.g., temperature, light) can be maintained. From a scientific point of view, these systems are well suited to investigate the influence of the studied treatment. Sulfur (S) is one of the indispensable macronutrients that is needed for optimal plant growth. However, lettuce (*Lactuca sativa* L.) has already been shown to respond significantly to different states of sulfur supply [2]. It might be, therefore, a good model plant. Generally, plants require S for the synthesis of a variety of metabolites such as the amino acids (e.g., cysteine and methionine), glutathione (GSH), certain vitamins, lipids, prosthetic groups, and further S-containing plant secondary metabolites [3]. In addition, numerous studies show that sulfur deficiency leads to poor quality and DM accumulation [4,5]. It was reported that sulfate deficiency leads to reduced synthesis of the enzyme Rubisco (ribulose-1,5-biphosphate carboxylase/oxygenase), which affects the assimilation rates of CO_2_, eventually leading to the delayed synthesis of carbohydrates, resulting in chlorosis of young leaves [6]. In particular, ATPS is involved in the abiotic stress tolerance of plants via various S-containing compounds, especially GSH, which functions as a storage and transport form of reduced S [7]. Because of the high reactivity of thiol (-SH) groups, S compounds, including Cys and GSH, are central to metabolic redox regulation [8]. The main induction of the GSH-based stress defense system, its role in ROS scavenging, and maintenance of cellular redox homeostasis were extensively demonstrated in plants under various environmental stresses [7,9]. The role of sulfur in reducing ROS-induced oxidative stress in plant cells is well documented [10,11,12,13].

Reactive oxygen species (ROS) are always occurring in cells. However, unless in a status of excess, they are indispensable, as they affect, for example, plant growth, reproduction, and photosynthesis. Due to abiotic conditions (e.g., enhanced ultraviolet radiation, drought), ROS levels can become imbalanced, which is commonly called oxidative stress. As a proof of concept, the analysis of the membranes’ lipid composition and oxidized lipids can provide an in-depth insight into the stress level of the plant. Especially the thylakoid membrane of the chloroplasts is particularly exposed to ROS due to the electron transport chain that occurs inside the membrane [14]. The accumulation of ROS, resulting from further biotic or abiotic stressors, leads to interactions with biomacromolecules such as proteins, lipids, or the DNA [15,16]. This can cause a disruption in the cellular structures and diminishing of the mechanisms behind, which is reflected in a reduction of the yield [17]. In the context of the thylakoids, ROS have a detrimental impact on several parts [18]. Next to the pigment–protein complexes [19,20,21], double bond-containing compounds (such as chlorophylls and unsaturated fatty acids in many lipids) as well as different enzymes and proteins of the photosystem II (PS II) were detected as targets of ROS [20,21,22].

Thylakoid membranes have a unique lipid composition. In addition to phosphatidylglycerols (PG), they contain three classes of glycolipids, namely, monogalactosyl- (MGDG), digalactosyl- (DGDG), and sulfoquinovosyldiacylglycerols (SQDG), which account for approximately 80–90% of membranes [23,24]. These glycolipids are mostly esterified with two different fatty acids, which can be widely varying in chain length and degree of saturation. Due to this wide range of possible combinations, a large number of individual glycolipids is possible, forming a complex membrane structure. As already mentioned, various stressors might lead to a reduction in the pigment content [23,24], a change in fatty acid composition, or a transformation/exchange of the different lipid classes [24,25,26,27,28]. In addition to SQDG, galactolipids represent the majority of the lipids in the thylakoid membrane and might be affected by S nutrition, especially S deprivation. This relationship between the influence of stressors such as S deficiency and the change in the different glycolipids and fatty acid distribution seems to be a powerful tool for estimating the stress level of plants. Additionally, a change in the degree of saturation and hydroxy-lation/oxidation by ROS seem to be possible as well. Oxidation by hydroxyl radicals could occur, affecting mainly polyunsaturated fatty acids (PUFA). The resulting products might serve as marker compounds for the stress level and might be influenced by S nutrition. The aim of this study was to investigate the influence of contrasting S levels on the distribution of the main thylakoid glycolipids and to determine lipid oxidation products in response to varied S levels, which can consequently illustrate a certain relationship. As already mentioned, lettuce might serve as a good model plant for sulfur-related stress. The comparison between red and green cultivars might enable us to differentiate between plants that differ in their endogenous composition of lipids, antioxidants, etc.

## 2. Results

### 2.1. The Effect of S Nutrition on Dry Matter Accumulation in Green and Red Lettuce

**Figure 1:**
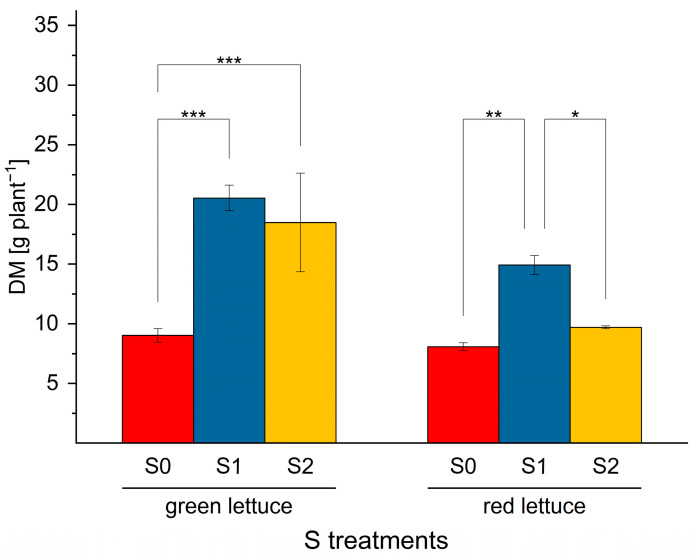
Dry matter (DM) accumulation in green and red multi-leaf lettuce grown in a hydroponic system under varied S levels (S0: 0 mM, S1: 1 mM, and S2: 1.5 mM, K_2_SO_4)_. Values represent the mean ± SD of independent replicates (*n* = 4). Asterisks indicate the different levels of significance; (* *p* ≤ 0.05; ** *p* ≤ 0.01; *** *p* ≤ 0.001); no asterisks mean there is no significance.

The findings of the study showed wide variations in DM accumulation in green and red multi-leaf lettuce under S nutrition. The data revealed that S sufficiency had a great influence on DM accumulation of both cultivars (Figure 1). Under S sufficiency (S1), a dramatic increase (*p* < 0.001) (by 127.8%) was observed in the green cultivar, whereas in red lettuce the DM was enhanced significantly (*p* ≤ 0.01) by 84% (Figure 1), suggesting that the green lettuce showed a greater performance in comparison to the red cultivar. Contrary to adequate S level (S1), S deficiency (S0 = 0) resulted in a significant decrease in DM accumulation in both cultivars (Figure 1). Owing to the importance of S as a crucial element required for plant growth and development, several studies reported adverse effects on lettuce biomass, including the yield and DM accumulation under S starvation [3,29,30]. Elevated S level (S2: 1.5 mM) enhanced significantly (*p* ≤ 0.001) (125.6%) the DM in green lettuce, whereas, in red ones, it increased significantly (by 67%) under higher S concentration.

### 2.2. Method Development for the Determination of Galactolipids

**Figure 2:**
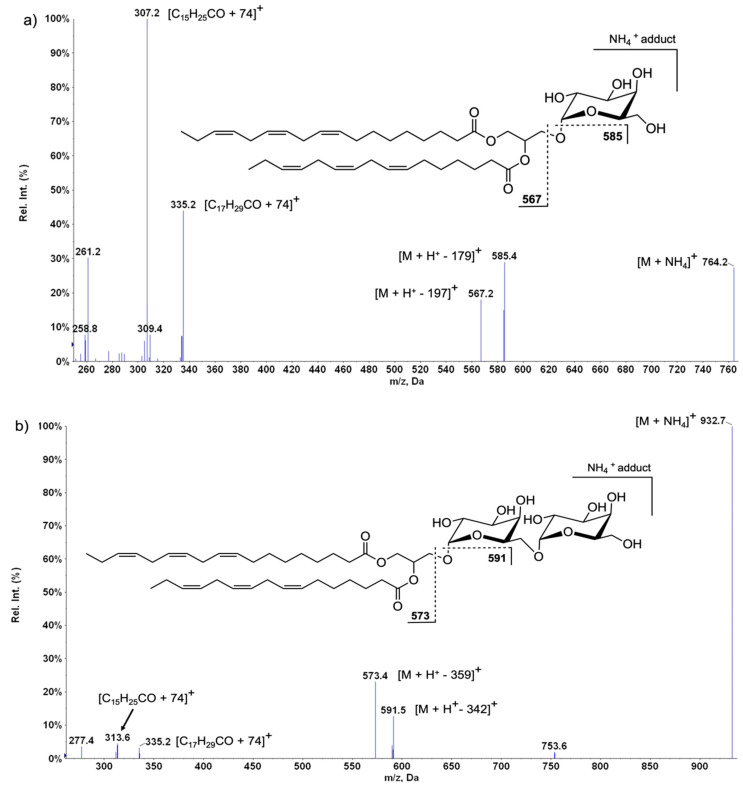
(**a**,**b**). LC-MS/MS fragmentation pattern of [M + NH_4_]^+^ ions of MGDG *m*/*z* 764 (**a**) and DGDG *m*/*z* 932 (**b**).

Table 1 and Table 2 summarize the precursor and fragment ions of all MGDG and DGDG determined in the present study. They were identified as [M+NH_4_]^+^ ions in the positive mode LC-MS spectra. Overall, eight molecular species of MGDG and eight DGDG were detected. Representative LC-MS spectra of MGDG and DGDG classes are shown in Figure 2. The typical fragmentation observed in the LC-MS/MS spectra of MGDG and DGDG species as [M+NH_4_]^+^ ions allows confirmation of the presence of these glycolipids. The LC-MS/MS spectrum of MGDG-764 (*m*/*z* 764) (Figure 2a) afforded the product ion at *m*/*z* 585.4 corresponded to [M+H^+^-179]^+^, resulting from the combined loss of NH_3_ (−17 Da) and the loss of a galactosyl moiety (−162 Da). It was formed by cleavage of the sugar hemiacetal oxygen bond with proton transfer to give a diacyl-glycerol structure. The second product ion observed at *m*/*z* 567.2 corresponded to [M+H^+^-197]^+^, which is formed by the combined loss of NH_3_ (−17 Da) and the loss of a hexose ring (−180 Da). Similarly, in the LC-MS/MS spectrum of DGDG-932 (*m*/*z* 932.6) (Figure 2b), losses of a carbohydrate moiety (loss of 180 + 162 Da and loss of 180 + 197 Da) were observed, each in combination with the loss of NH_3_ (−17 Da), resulting in the formation of product ions at *m*/*z* 573.4 [M+H^+^-359]^+^ and 591.5 [M+H^+^-342]^+^. In addition, the product ions that can be used to deduce the composition of the acyl chains of the esterified fatty acids were detected. These ions resulted from each fatty acyl group as acylium ion (RCO + 74 Da).

These ions were delivered at *m*/*z* 305.2 and 333.2 in the MGDG spectrum (Figure 2a) and corresponded to the fatty acids 16:3 and 18:3, respectively. In the DGDG spectrum (Figure 2b), the [RCO + 74]^+^ ions were obtained at *m*/*z* 313.6 and 335.2 and corresponded to the fatty acids 16:0 and 18:3, respectively [31,32].

### 2.3. Method Validation for the Determination of Galactolipids

The method for the simultaneous determination of MGDG and DGDG was validated in terms of linearity, precision, accuracy, limit of detection (LOD), and limit of quantification (LOQ) according to well-accepted guidelines [33]. The determination coefficients (R^2^) of the calibration curves showed an excellent correlation with high mean values (0.998  ±  0.001) for MGDG and DGDG (0.9963 ± 0.004). According to the guidelines of the US Food and Drug Administration (FDA), the acceptable limit of coefficient of variation (CV) is taken to be < 15%. Methods with a CV < 15% can be accurate and precise at the same time [33]. The calculated CV for the external standards (*n* =  4) ranged between 1.7 and 4.34%, indicating that the method meets the FDA’s requirements in terms of precision. The LOQ determined for the quantitation of MGDG was 0.201 ± 0.063 μg mL^−1^. Whereas the LOQ determined for the quantitation of DGDG was 0.184 ± 0.059 μg mL^−1^.

### 2.4. SQDG in Lettuce Plants

**Figure 3:**
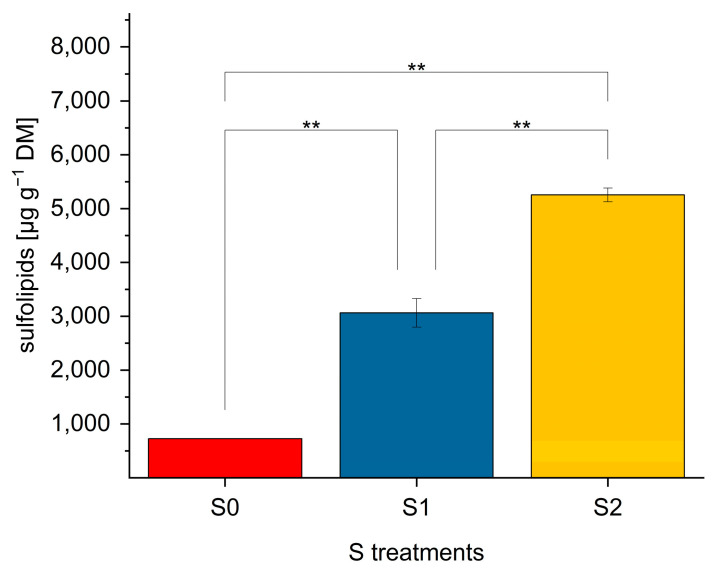
Total sulfolipid content [µg g^−1^ DM] in red multi-leaf lettuce grown under different S treatments. Values represent the mean ± SD of independent replicates of lettuce treated with S0: 0 mM (*n* = 1), S1: 1 mM (*n* = 5), and S2: 1.5 mM (*n* = 2) K_2_SO_4_, respectively. Asterisks indicate the different levels of significance; (** *p* ≤ 0.01); no asterisks mean that there is no significance.

Total SQDG contents were calculated from the sum of all individual SQDG. Total SQDG are illustrated in Figure 3. With the aid of the synthesized internal standard SQDG-846, all other single SQDG were semi-quantitatively determined.

SQDG content significantly differed in lettuce plants grown under various S levels. In comparison to green multi-leaf lettuce a dramatic increase in SQDG concentration was detected following S fertilization in red multi-leaf lettuce [24]. For instance, a significant 7.2-fold increase (from 0.73 to 5.3 mg g^−1^ DM) was observed between S0 and S2 levels (*p* < 0.01). Similar to the green multi-leaf lettuce, a further increase was found at elevated S level, compared to an adequate S supply.

With regard to the (individual) SQDG composition of the samples from the different S treatments, 22 single SQDG were detected (Table 3). To determine the formation of each individual SQDG, an enrichment factor was calculated. This factor can display whether an individual SQDG is preferentially formed by S fertilization. For this purpose, the general factor (calculated from the increase in the total concentration of S0 to S2 levels) is compared with the individual factors. The mean enrichment was 7.2 (5.3 mg g^−1^ DM/0.73 mg g^−1^ DM). The following SQDG were the most enriched derivates (fatty acids linked to glycerol are presented in the brackets): SQDG-787 (16:3/16:0), SQDG-813 (18:3/16:1), SQDG-819 (18:1/16:0), SQDG-837 (18:3/18:3), and SQDG-845 (18:1/18:1). SQDG-765 (14:0/16:0), SQDG-793 (16:0/16:0), SQDG-867 (14:0/16:1), SQMG-555 (16:0), SQDG-831 (16:0/18:3-OH), and SQDG-833 (16:0/18:2-OH) had enrichment factors lower than 7.2. A similar trend in green multi-leaf lettuce was found to that of red multi-leaf lettuce [24].

**Figure 4:**
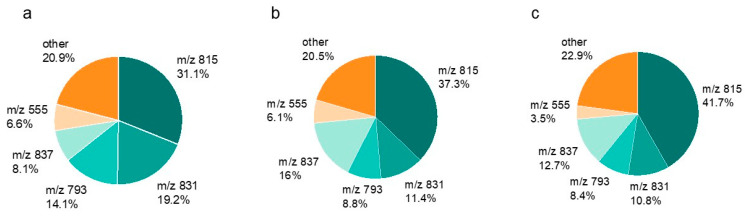
Distribution of the most abundant sulfoquinovosyl diacylglycerol derivatives (SQDG) (%) in relation to total SQDG content in red multi-leaf lettuce. Plants were treated with (**a**) S0: 0 mM, (**b**) S1: 1 mM, and (**c**) S2: 1.5 mM K_2_SO_4_. Individual SQDG were displayed with their mass to charge ratio (*m*/*z*).

SQDG-815, SQDG-831, SQDG-793, SQDG-837, and SQDG-555 were the most abundant sulfolipids in the membrane of red multi-leaf lettuce (Figure 4). As in the green multi-leaf lettuce, SQDG-815 accounted for up to 42% (in sulfur level S2). These five SQDG represented around 80% of total sulfolipid content.

#### Oxidized SQDG in Lettuce Samples

ROS are produced by photosynthesis as a side effect. Subsequently, the content of these species is balanced in the cells by diverse scavenging processes of the endogenous antioxidant system; nevertheless, an imbalance of the ROS concentration can be induced by biotic or abiotic influences and lead to oxidation of biomolecules such as lipids [34]. Especially the double bonds of PUFA are affected by oxidation. Consequently, the resulting products are hydroxy fatty acids (LOH) [34].

**Figure 5:**
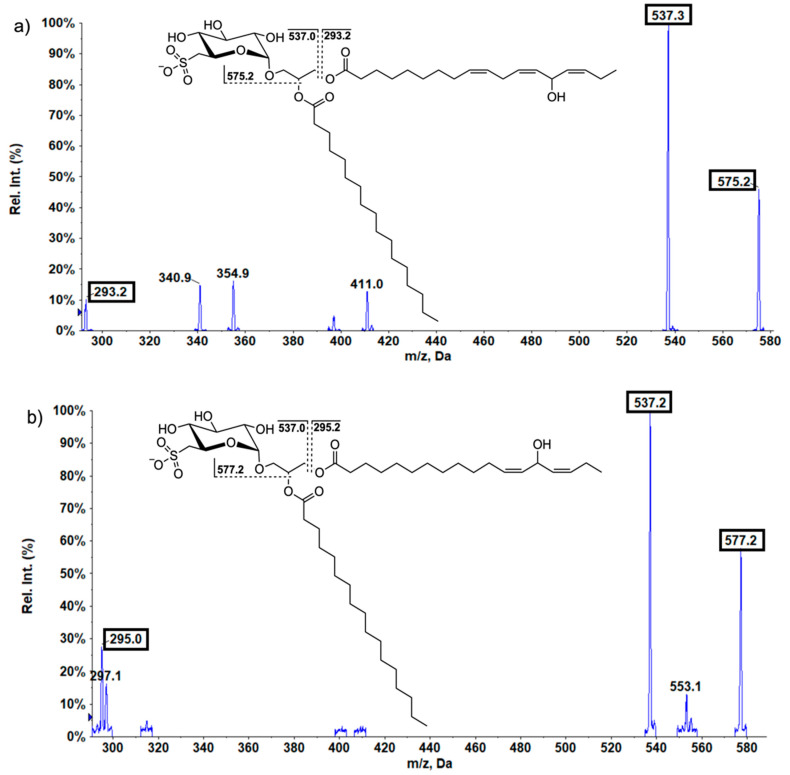
(**a**,**b**). Lipid fragments of the fragmentation patterns of the sulfoquinovosyl diacylglycerol derivative (**a**) SQDG-831 and (**b**) SQDG-833; Rel. Int.: relative intensity.

**Figure 6:**
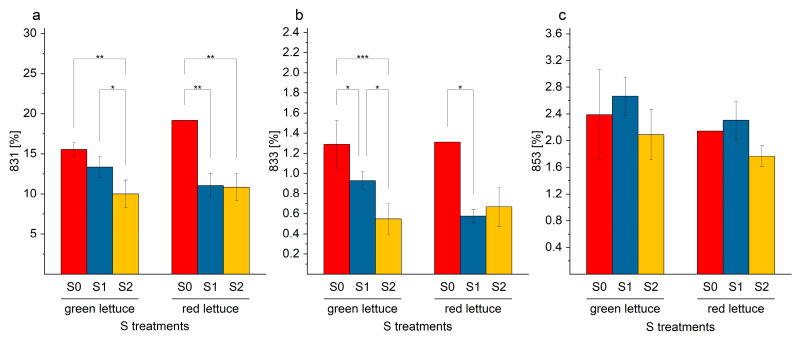
(**a**–**c**). Contents (%) of individual hydroxylated sulfoquinovosyl diacylglycerol derivatives, (**a**) SQDG-831; (**b**) SQDG-833; and (**c**) SQDG-853, in green and red multi-leaf lettuce grown under different S treatments. Values represent the mean ± SD of independent replicates of lettuce treated with S0: 0 mM (*n*_green leaf_ = 4; *n*_red leaf_ = 1), S1: 1 mM (*n*_green leaf_ = 6; *n*_red leaf_ = 5), and S2: 1.5 mM (*n*_green leaf_ = 5; _nred leaf_ = 2) K_2_SO_4_, respectively. Asterisks indicate the different levels of significance (* *p* ≤ 0.05; ** *p* ≤ 0.01; *** *p* ≤ 0.001); no asterisks mean that there is no significance.

In the analyzed lettuce samples, the oxidized SQDG (SQDG-OH) could be detected. The SQDG were first identified by their specific fingerprint fragments (*m*/*z* 81, *m*/*z* 165, and *m*/*z* 225) [35]. Additionally, specific fragments of hydroxy fatty acids (*m*/*z* 293; 18:3-OH, *m*/*z* 295; 18:2-OH) allowed direct characterization of individual oxidized SQDG. A representative example of the specific fragmentation pattern for oxidized SQDG is shown in Figure 5a,b. Based on these hydroxy fatty acids fragments, the oxidized species SQDG-831, SQDG-833, and SQDG-853 were detected. SQDG-OH (*m*/*z* 831), the most common SQDG-OH, is the oxidized linolenic acid (18:3-OH) equivalent of SQDG-815. SQDG-833 is the LOH equivalent to SQDG-817, and SQDG-853 is equivalent to SQDG-837. A preferential oxidation of SQDG-837 is most likely due to the two attached PUFA (18:3/18:3). The decrease in the enrichment factors of SQDG-OH leads to the assumption that these are formed preferentially under S deprivation conditions. With regard to SQDG-831 and SQDG-833, a clear decrease in the percentage distribution in the membrane is visible (Figure 6). This decrease is further demonstrated by the enrichment factor. When the concentrations in the different S supply levels were not altered, the enrichment factor is one. However, there were enrichment factors of 4.1 and 3.7 (SQDG-831 and SQDG-833), which indicated that the concentrations of these individual SQDG were enriched; however, the concentration has increased less than the total SQDG concentration. The total SQDG concentration in plants for S2 level was 7.2 times higher than the total content in S0 level (enrichment factor 7.2). In summary, the concentration of all detected SQDG-OH were decreased by S nutrition in lettuce.

**Figure 7:**
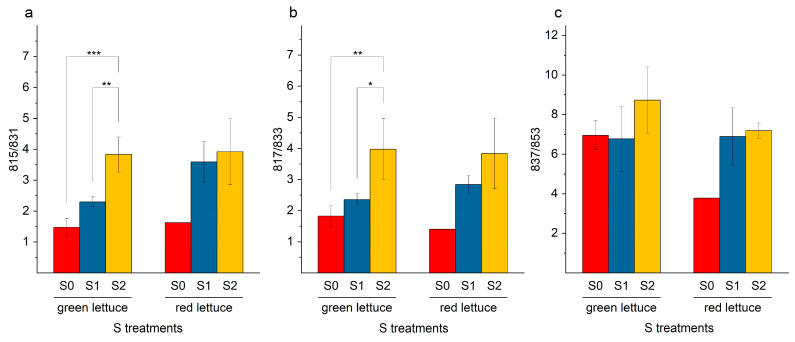
(**a**–**c**). Ratios of individual sulfoquinovosyl diacylglycerol derivatives and the corresponding hydroxylated sulfoquinovosyl diacylglycerol derivatives, (**a**) 815/831, (**b**) 817/833, and (**c**) 837/853, in green and red multi-leaf lettuce grown under different S treatments. Values represent the mean ± SD of independent replicates of lettuce treated with S0: 0 mM (*n*_green leaf_ = 4; *n*_red leaf_ = 1), S1: 1 mM (*n*_green leaf_ = 6; *n*_red leaf_ = 5), and S2: 1.5 mM (*n*_green leaf_ = 5; *n*_red leaf_ = 2) K_2_SO_4_, respectively. Asterisks indicate the different levels of significance (* *p* ≤ 0.05; ** *p* ≤ 0.01; *** *p* ≤ 0.001); no asterisks mean that there is no significance.

When considering the ratios of the SQDG and their corresponding LOH equivalents, it was noticeable that the ratio significantly increased in case of the pairs 815/831 (Figure 7a) and 817/833 (Figure 7b). This could be attributed to the enrichment factors. The SQDG-OH were decreased in concentration, while SQDG was increased or accumulated under adequate or elevated S levels.

### 2.5. Galactolipids in Lettuce Samples

**Figure 8:**
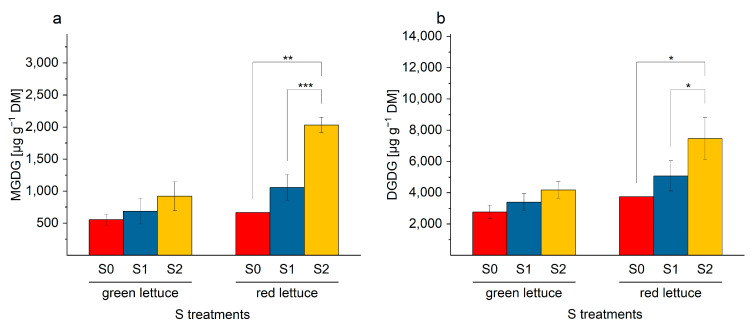
(**a**,**b**). Total contents [µg g^−1^ DM] of (**a**) monogalactosyl diacylglycerol (MGDG) and (**b**) digalactosyl diacylglycerol (DGDG) in green and red multi-leaf lettuce grown under different S treatments. Values represent the mean ± SD of independent replicates of lettuce treated with S0: 0 mM (*n*_green leaf_ = 4; *n*_red leaf_ = 1), S1: 1 mM (*n*_green leaf_ = 6; *n*_red leaf_ = 5), and S2: 1.5 mM (*n*_green leaf_ = 5; *n*_red leaf_ = 2) K_2_SO_4_, respectively. Asterisks indicate the different levels of significance (* *p* ≤ 0.05; ** *p* ≤ 0.01; *** *p* ≤ 0.001); no asterisks mean that there is no significance.

Similar to SQDG, the total content of galactolipids showed an increase under higher S fertilization. The results of the total galactolipid contents were illustrated in Figure 8. In plants grown under S2 condition, the highest contents of both galactolipids were detected, especially in red-multi leaf lettuce. In comparison to the MGDG concentrations in lettuce plants grown under S0 and S2 levels, a 1.8-fold and a 3.1-fold increase were detected in green and red multi-leaf lettuce, respectively. The DGDG concentration was enriched by S fertilization, for instance, a 1.5-fold and 2-fold increase was determined in both lettuce cultivars. In the red multi-leaf lettuce plants, the MGDG and DGDG were enhanced significantly. The increase in the total galactolipid concentration was represented by the ratio of the concentration in S2 and S0, demonstrating that MGDG is formed more than DGDG in response to high amounts of S in the medium (S2). However, in red multi-leaf lettuce, the content was more enriched in comparison to the green multi-leaf lettuce plants. The different increase in the content of MGDG and DGDG is also represented by the ratio of the two concentrations to each other. The ratio of DGDG/MGDG changed from 5.4 or 5.6 in S0 to 4.5 or 3.7 in S2 in green and red lettuce, respectively. The higher change in the ratio in red lettuce reflects the stronger influence of S on the MGDG concentration.

Individual MGDG and DGDG were identified and quantified with the aid of a commercial MGDG and DGDG mix on the basis of retention time and *m*/*z* fragments. Collectively, the percentage occurrence of seven MGDG and seven DGDG was evaluated. The change in the individual content in response to the varied S levels is presented in Table 4.

**Figure 10:**
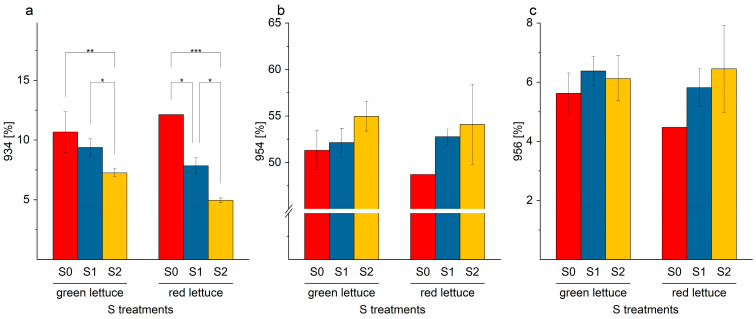
(**a**–**c**). Content (%) of individual digalactosyl diacylglycerol derivates (**a**) DGDG-934; (**b**) DGDG-954; and (**c**) DGDG-956, in green and red multi-leaf lettuce grown under different S treatments. Values represent the mean ± SD of independent replicates of lettuce treated with S0: 0 mM (*n*_green_ = 4; *n*_red_ = 1), S1: 1 mM (*n*_green_ = 6; *n*_red_ = 5), and S2: 1.5 mM (*n*_green_ = 5; *n*_red_ = 2) K_2_SO_4_, respectively. Asterisks indicate the different levels of significance (* *p* ≤ 0.05; ** *p* ≤ 0.01; *** *p* ≤ 0.001); no asterisks mean that there is no significance.

Similar to the SQDG derivatives, similar trends of an increase or a decrease in individual galactolipids were identified. Individual MGDG or DGDG with saturated or monounsaturated fatty acids decreased (MGDG-770: 18:3/16:0; DGDG-934: 18:1/16:1; Figure 9b and Figure 10a). In contrast, galactolipids with attached PUFA were preferentially formed (MGDG-794: 18:2/18:3, and DGDG-954: 18:3/18:3; DGDG-956: 18:2/18:3, Figure 9a and Figure 10b,c).

### 2.6. Determination of the Antioxidant Capacity in Lettuce Samples

Antioxidants can slow down or prevent the oxidation of cell membrane components such as the PUFA in the glycolipids. Under stress conditions, they were required in larger quantities to prevent the plant damage. Typically, antioxidants are chemical structures with aromatic rings (‘phenolic compounds’) that can scavenge radicals. The trends in the total phenolic content (TPC) in green and red multi-leaf lettuce are presented in Table 5. The application of S led to average to lower TPC values compared to S deprivation (level S0). Red lettuce under S2 level had a higher TPC than green lettuce grown under S deprivation (S0). Adequate and higher S levels (S1 and S2) had similar impacts in green and red lettuce (total decrease of 36% or 32% TPC, respectively). However, the differences in TPC attributed to the different S levels were statistically not significant. The Trolox equivalents antioxidant capacity (TEAC) assay is an easy assay to determine hydrophilic and lipophilic antioxidants. The ABTS^•+^ reacts quickly with antioxidants [36]. Trends in the antioxidant capacity are similar to the trends observed for the TPC. Higher values were determined in lettuce plants under stress condition (S0). Overall, red lettuce showed a more intense reaction towards ABTS^•+^ compared to green lettuce (Table 5). This was expected, as the red leaf color already indicated a higher phenolic content. A significant decrease in the TEAC values was detected in red lettuce from S0 to S1 levels.

## 3. Discussion

Sulfur deprivation is known to affect biomass, overall morphology, and the nutritional value of plants. Moreover, S plays an important role in the protection of plants from environmental stresses through its antioxidant protective functions [11,37,38]. In the current study, green and red lettuce cultivars were grown in a hydroponic system and treated with different S fertilization levels (S0: 0 mM, S1: 1 mM, and S2: 1.5 mM). Since both pigmented cultivars will have distinct physiological responses under stress conditions (S-deficiency). The green and red lettuce were selected to provide an in-depth understanding of their differential responses to obtain the most stress-tolerant cultivar with higher antioxidant capacity by investigating glycolipids of the thylakoid membrane. A contemporary study indicated that red lettuce has a better adaptive response to nutrient starvation in comparison to the green cultivar [39].

Consequently, the aim of the present study was to determine the influence of S nutrition and especially S-related stress on the content and composition of the three major glycolipid classes, SQDG, MGDG, and DGDG. These lipids are especially exposed to oxidative stress because of their localization in the thylakoid membrane and binding to the photosystems (PS). Therefore, the focus was additionally on the analysis of the modified lipids due to the presence of oxidized fatty acids.

The increase in S supply enhanced the accumulation in the total SQDG content in the red lettuce similar to the green lettuce analyzed in a contemporary study [26]. Both total SQDG contents, as well as individual SQDG with attached PUFA, were increased owing to the higher S supply.

Lettuce plants treated with elevated S levels exhibited a higher ratio of SQDG of the total membrane lipids in comparison to the plants grown under S deprivation. A reason for the higher increase in SQDG could be attributed to the induction of SQDG biosynthesis, when S is not required for the synthesis of other S-containing molecules [26].

However, total MGDG and DGDG contents were also enhanced in lettuce exposed to higher S levels. It was discussed previously that the alteration of lipid biosynthesis can influence plant growth and development [40,41,42]. With regard to chloroplast membranes in lettuce plants grown under S deprivation, this might result in a modification of the membrane abundance and fluidity demonstrated by lower contents of total MGDG, DGDG, and SQDG and by a higher degree of saturation of the fatty acids. Especially the thylakoid membrane is important in the context of photosynthesis. Additionally, in the electron transport chain, different proteins and complexes are embedded in this membrane. Moreover, lipids are involved in the formation of the membrane in form of a bilayer and in the stacking of proteins. Different glycolipids were identified to be part of protein–cofactor complexes in the thylakoid membrane of higher plants and bacteria [43,44,45,46]. For example, SQDG contributes to the structural and functional integrity of the PS II [47,48]. Therefore, SQDG synthesis was enhanced in S starvation for stabilizing the complex [49].

In addition to PS II, SQDG is also associated with the Cyt b_6_/f complex. However, SQDG could still be detected in a significantly lower amount in the studied red lettuce plants cultivated under S deprivation. Nevertheless, the most important functions including the stabilization of the PS II and the organization and assembly of the Cyt b_6_/f complex could be maintained. Likewise, galactolipids may partly take over the structural and functional tasks of the SQDG as both are the main membrane constituents. A decrease in the lipid contents could also affect the chloroplast function, probably the lamellar structure and the stacking of the proteins. In addition to S deficiency, other nutrient deficiencies (e.g., nitrogen) lead to a reduction in the galactolipid content, indicating that photosynthetic units decrease during nutrient stress and negatively affect the thylakoid membrane abundance [50]. Photosynthetic membrane lipids are associated with the plant’s response to abiotic stress by maintaining the structure of the chloroplast. In this context, they are involved in the individual processes of preserving the lamellar structure of grana, stabilizing chloroplast membranes [51], facilitating the stacking of proteins in the membrane [50,52,53], and adjusting the degree of fatty acid desaturation for regulating membrane fluidity [54,55,56,57,58]. The increased ratio of galactolipids in the lettuce membrane grown under S-deficiency might be a mechanism of plants’ acclimation responses under stress conditions. As previously mentioned, an increased DGDG/MGDG ratio was for abiotic stress situations induced by salt stress, drought, or heat stress [59,60]. The enhanced ratio could be related to the necessity of plant need to an additional membrane stabilization by upregulation of DGDG synthesis. Because of the smaller head group of MGDG, only inverted hexagonal II structures and non-bilayers can be formed, while in contrast, DGDG is able to form regular bilayers [61]. Therefore, the ratio between DGDG and MGDG is crucial for the structure of the membrane. Especially, the change in MGDG content can facilitate the proper stacking and development of thylakoid membranes and proteins, resulting in the maintenance of intact chloroplast structure under abiotic stress [40]. Additionally, DGDG seem to be more important to mitigate the photoinhibition of PS II by reducing the ROS production and photooxidative damage [59]. Similar results were observed in the present study. In lettuce grown under S deprivation conditions, DGDG/MGDG ratio increased likewise. This might be a mechanism that can enable plants to cope with nutritional stress situations induced by S starvation. In plants that were adequately or excessively supplied with S, the ratio was decreased and MGDG were formed more preferably Under both adequate and elevated S levels, a significant increase in galactolipid content was detected. Comparatively, high galactolipid content seems to improve adaption towards, e.g., heavy metal stress. Zhang et al. [32] reported that by overexpression of the rice *MGD* gene (*OsMGD*), the new growth of the roots in aluminum stress was faster than in the wildtype. Additionally, aluminum caused less lipid peroxidation and damage to the membrane integrity in the overexpressing rice mutants.

In the present study, the total content of the different lipid species differed between the lettuce cultivars. In addition to the fact that the contents were not comparable between the cultivars, trends in the individual lipids were quite similar.

Under S deprivation, the lettuce plants synthesized a large number of individual lipids with a lower degree of unsaturation of the fatty acids. This was expressed by, e.g., the metabolites SQDG-765 and SQDG-793 with at least one saturated fatty acid (14:0 or 16:0) or, e.g., palmitoleic acid (16:1) or oleic acid (18:1) attached. This trend was visible in red as well as green lettuce [24]. Another common feature in lettuce grown under S deficiency is that higher ratios of individual LOH were detectable. The modification of lipids is possible by enzymatic or non-enzymatic reactions with hydroxyl or hydroperoxyl radicals, singulett oxygen, or lipoxygenases. They initiate lipid peroxidation and lead to primary and secondary products. The radicals and singulett oxygen are characterized by a high reactive power; both belong to the ROS. Under very low levels, ROS are necessary as they act as signaling molecules. By an increased ROS formation and an imbalance of ROS-scavenging mechanism, resulting from, e.g., different environmental stresses, the reactive molecules can modify biomolecules (i.e., lipids, proteins, and DNA) with a loss of function [9,62,63,64]. The oxidation of lipids, which are a part of the thylakoid membrane, seems to be more presumably, because of a direct proximity of the electron transport chain. Therefore, SQDG seem to be frequently subjected to a hydroxylation by ROS. To the best of our knowledge in both lettuce cultivars, LOH attached to selected SQDG were detected for the first time. The following SQDG-OH could be identified: SQDG-831, SQDG-833, and SQDG-853. The fragment with the *m*/*z* of 293 consists of the fatty acid 18:3 (277.4 g mol^−1^), which is negatively charged, plus a hydroxy group (16 g mol^−1^). By the typical SQDG fragments *m*/*z* 81, *m*/*z* 224.7, and *m*/*z* 164.7, it can be confirmed that the present molecule is a sulfoquinovosyl diacylglycerol derivative [65]. This modified SQDG could be a new marker molecule for a higher abiotic stress level (and associated higher ROS formation) in plants. Especially SQDG-831 was detectable in high amounts in the lettuce plants under S deprivation. SQDG-831 consist of an oxidized linolenic acid (18:3-OH) and are the oxidized equivalent of SQDG-815, one of the most common sulfoquinovoses in plants. As SQDG contents vary in different plant species, the SQDG-815/SQDG-831 ratio could be used for this purpose. Thus, larger ratios can be associated with lower stress levels, while lower ratios might be an indication for an increased oxidation of SQDG in the cell and thus, a higher stress level. In both S-deprived lettuce cultivars, ratios of approximately 1.5 were calculated. In contrast, this ratio increased significantly (*p* < 0.01) in green lettuce depending on the S fertilization. Similar trends were visible in red lettuce; however, they were not significant. Pospíšil et al. summarized in a quite comprehensive way the formation of lipid peroxidation products [15]. LOH have a comparatively low interaction capability [15], which probably leads to their easier detection. For example, the LOH hydroxyocatdecatrienoic acid with its different isomers was identified in *Arabidopsis thaliana* [35].

In plant cells, chloroplasts are especially exposed to ROS [14], enabling a higher chance for the present lipids to be more frequently affected than other organelles. The formation of lipid radicals was detected especially in PS II membranes [66]. As SQDG are associated with the PS II, they might undergo lipid peroxidation more frequently. The hypothesis of a higher ROS level resulting from a higher nutritional stress in plants grown in response to S limitation was supported by the results of the increased antioxidant capacity and TPC. The antioxidant molecules, such as phenolic compounds, are involved in ROS scavenging capacity and can be interpreted to be a part of the defense mechanism of the lettuce plants exposed to the S limiting condition. This is a common stress response to biotic and abiotic stress [67]. Gershenzon [68] reported similar effects in plants exposed to different nutrient deficiency situations. Starvation stimulates the biosynthesis of phenolic compounds. For instance, flavonoids are involved in the scavenging of ROS, in addition to the protection of the incoming radiation and the quenching of radicals created by stress [69]. The enhanced antioxidant capacity or a higher TPC can protect the cells from the higher ROS level. The promising antioxidant capacity and the TPC act as indicators of an enhanced stress potential and an accompanying reaction in the plants under S deprivation. In the Achillea species, higher TPC levels were determined when the plants experienced drought and salt stress [70]. Nutritional stress such as S deprivation had a similar outcome in the two analyzed lettuce cultivars.

In contrast, under adequate or excess S treatments, lower levels of antioxidants were detected, which is correlated with lower levels of ROS. The lower stress levels were illustrated by the degree of unsaturation of the fatty acids of the glycolipids. Overall, a higher percentage of lipids with higher amounts of linked PUFA were detected. Individual lipids, including DGDG, MGDG, or SGDG, were formed preferentially if unsaturated fatty acids were attached. In particular, MGDG-794, DGDG-954, and SQDG-837 consist of highly unsaturated fatty acids and were found in the highest ratios in lettuce plants when exposed to excess S level. An increase in the overall PUFA content in plants is known as an indicator of a better acclimation to various abiotic stress conditions.

## 4. Materials and Methods

### 4.1. Plant Material and Growth Conditions

The hydroponic greenhouse experiment was conducted at the Institute of Plant Nutrition and Soil Science, University of Kiel, Kiel, Germany, as previously described by Körber et al. (2022) [24]. The green ‘Hawking RZ’ and red ‘Barlach RZ’ lettuce cultivars were used exemplarily in this study. Seeds were planted in a growth chamber between two filter papers in sandwich blots moistened with 1:5 diluted 10 mM CaSO_4_ solution. After 14 d, seedlings were transferred to 10 L-black containers (two per pot) and cultivated in a greenhouse under inductive environmental conditions (18/14 °C day/night cycle and a 14 h day-1 photoperiod). The nutrient solution was composed of the following nutrients: 2 mM Ca(NO_3_)_2_, 0.5 mM NH_4_H_2_PO_4_, 0.5 mM MgCl_2_, 2 mM KNO_3_, in addition to a micronutrient solution of 60 μM Fe-EDTA, 10 μM H_3_BO_3_, 2 μM MnSO_4_, 0.5 μM ZnSO_4_, 0.3 μM CuSO_4_, and 0.01 μM (NH_4_)_2_Mo_7_O_24_. The nutrient solution was changed out once a week. Additionally, the pH was checked regularly and adjusted from 6.0 to 6.5. To investigate the differential response of both pigmented lettuce cultivars with regard to changes in selected galactolipids of the thylakoid membranes under S deficiency, sufficiency, and elevated levels, three varying S levels in the form of potassium sulfate (K_2_SO_4_) were selected as follows: (I) The control (S0: 0 mM), (II) Adequate S level (S1: 1 mM), and (III) Higher S level (S2: 1.5 mM). The treatments were applied in the nutrient solution. KHCO_3_ was added to balance the K ion concentration in the nutrient solution among the three S levels. For S1, 2 mM KHCO_3_ and for S2, 3 mM KHCO_3_ were used.

Additionally, four replicates were assigned in a completely randomized design. Fifty-five days after transplanting the seedlings, plants were harvested, and the lettuce leaves were washed with distilled water and dipped into liquid nitrogen to facilitate better drying using the freeze dryer (Gamma 1–20, Martin Christ Gefriertrocknungsanlagen GmbH, Osterode am Harz, Germany). The dry matter (DM) of the leaves was determined. Subsequently, dried leaves were milled into a fine powder and kept for the analyses of the galactolipids.

### 4.2. Chemicals and Materials

The galactolipid standards were purchased from Avanti polar Lipids Inc. (Alabaster, AL, USA). Acetonitrile, chloroform, and methanol were purchased from Carl Roth GmbH and Co KG (Karlsruhe, Germany). Ammonium acetate, copper sulfate, and potassium chloride were obtained from Sigma-Aldrich GmbH (Munich, Germany). Additionally, ammonia (25%) and nitric acid (65%) were acquired from Merck KGaA (Darmstadt, Germany). All aqueous solutions were prepared with deionized water, generated by a Purelab flex water purification system (VeoliaWater Technologies Deutschland GmbH, Celle, Germany). Amino-phase (NH_2_) solid phase extraction cartridges (6 mL, 500 mg) were purchased from Macherey-Nagel GmbH and Co. KG (Düren, Germany).

### 4.3. Preparation of Internal SQDG Standard

The internal standard (2-*O*-hexadecanoyl-*d*_31_)-1-*O*-(9Z,12Z,15Z-octadecatrienoyl) glycerol 3-(6-deoxy-6-sulfo-β-d-glucopyranoside) (IS; SQDG-846; *m*/*z* 846) for the quantification of the sulfolipids was prepared according to Sitz et al. [71], with slight modifications. For the modified preparation of the internal standard, the precursor (3-O-[(2′,3′,4′-tri-O-levulinyl-6′-thioacetyl)-β-d-glucosyl]-(R/S)-glycerol) was esterified successively with α-linolenic acid and palmitic acid-*d*_31_ and the oxidation was performed with H_2_O_2_ instead of oxon^®^. The IS was purified by preparative HPLC on a C_18_ column (10 μm, 250 mm × 22 mm) with acetonitrile:water (35:65, 2 mL min^−1^).

### 4.4. Lipid Extraction Procedure

Galactolipids and sulfolipids were extracted with a chloroform/methanol (3/2, *v*/*v*) mix according to the method described by Fischer et al. [35]. Extraction from the lettuce samples was improved by a ball mill (frequency 25 Hz, 7 balls ø = 1.5 cm; Retsch MM 400, Retsch GmbH, Haan, Germany).

### 4.5. Separation and Purification of the Lipids by Solid Phase Extraction

Separation of the chargeless galactolipids and negatively charged sulfolipids was achieved by a solid phase extraction (SPE) with anion exchange function, following a protocol described by Fischer et al. [35], with slight modifications. NH_2_-SPE cartridges were used for separating the different charged lipids. The extracted sample was loaded onto the cartridge. Galactolipids were eluted with 10 mL chloroform/methanol (9/1, *v*/*v*) and 10 mL chloroform/methanol (5/5, *v*/*v*) and collected in a 50 mL-tube (‘fraction 1’). SQDG (‘fraction 2’) were eluted with 10 mL chloroform/methanol (4/1, *v*/*v*) containing 100 mM ammonium acetate and 2% NH_3_. Fraction 2 was mixed with 2 mL 0.9% potassium chloride solution. After vortexing, ultra-sonification for 10 min, and centrifugation (10 min, 12,000× *g*), the organic phase was separated. Solutions were dried with a gaseous stream of nitrogen, and fraction 1 was resolved in 3 mL methanol (‘solution 1’ for the determination of MGDG and DGDG), and fraction 2 was resolved in 1 mL MeOH (‘solution 2’ for the determination of SQDG).

### 4.6. Determination of Sulfolipids Using LC-ESI-MS/MS

Mass spectrometer parameters were used according to Fischer et al. [35], with minor modifications. LC-MS analysis was performed on an Agilent 1260 Infinity II HPLC-system (Agilent Technologies Deutschland GmbH, Waldbronn, Germany) coupled to a 5500 QTrap triple quadrupole MS/MS system (AB Sciex Germany GmbH, Darmstadt, Germany). Separation of ‘solution 2’ (the SQDG fraction) was achieved with a Kinetex^®^ C18 column (5 µm, 100 Å, 150 mm x 2.1 mm; Phenomenex Ltd., Aschaffenburg, Germany), using a constant flow rate of 0.3 mL min^−1^. Eluent A was water and eluent B was acetonitrile/water (9/1; *v*/*v*), each with 10 mM ammonium acetate. The elution started with 3% eluent B for 2 min and linearly increased to 99% eluent B within 4 min, which was kept constant for 32 min. Then, the composition was readjusted to 3% eluent B within 2 min, followed by 4 min of re-equilibration. The SQDG derivative *m*/*z* 846 was used as internal standard in a concentration of 0.5 µg·mL^−1^.

### 4.7. Quantification of Galactolipids (MGDG, DGDG) by LC-ESI-MS/MS

Galactolipids such as monogalactosyldiacylglycerols (MGDG) and digalactosyldiacylglycerols (DGDG) are crucial lipids involved in photosynthesis and metabolic regulation. They are affected by various stress conditions such as nutrient limitation. Often when galactolipids are quantified, it is only performed as a sum parameter using methods that do not differentiate between the individual MGDG and DGDG. However, to obtain an impression of the distribution and to evaluate whether the formation of individual MGDG and DGDG changes depending on sulfur nutrition, it was necessary to develop a method that simultaneously determines individual MGDG and DGDG. The development of the new LC-ESI-MS/MS method, as well as the method validation process, are based on procedures described by Fischer et al. [35] with slight modifications. The galactolipids MGDG and DGDG were analyzed on an Agilent 1260 Infinity Quarternary LC System (Agilent Technologies Deutschland GmbH, Waldbronn, Germany) coupled to a triple quadrupole API 4000 QTrap mass spectrometer (Sciex Germany GmbH, Darmstadt, Germany) equipped with a turbo spray source, which was operating in positive ion mode, with the following mass spectrometer settings: ion spray voltage: 4500 V; ion source heater  =  650 °C source gas 1: 50 psi; source gas 2: 0 psi; and curtain gas: 10 psi. The injection volume for all samples was 5 μL, the column oven temperature was set to 20 °C, and the autosampler was maintained at 4 °C. The separation of analytes was achieved using a Kinetex^®^ C18 column (2.6 μm, 150 mm × 2.1 mm), equipped with a Kinetex^®^ C18 security guard column (Phenomenex Inc., Torrance, CA, USA), using a constant flow rate of 300 μL min^−1^. Eluent A was water with 2 mM ammonium acetate and eluent B was acetonitrile. The elution started with 55% eluent B for 2 min and linearly increased to 87.5% eluent B within 2 min, which was kept constant for 41 min. Then, the composition was readjusted to 55% eluent B within 1 min, followed by 4 min of re-equilibration. MRM transitions of the different galactolipids were obtained by direct flow injection of the MGDG and DGDG mix (in methanol with 2 mM ammonium acetate) into the ESI source in positive mode. Each glycolipid (MGDG, DGDG) precursor ion was determined based on three specific fragment ions (product ions). Table 1 and Table 2 summarize the precursor and fragment ions of all compounds tested in the present study. Mass analyzer settings were optimized for all analytes to maximize the transmission and sensitivity of each characteristic mass transition. These optimizations were acquired automatically by using autotune mode provided by the Analyst^®^ software 1.6.1 (AB Sciex Germany GmbH, Darmstadt, Germany).

### 4.8. Method Validation

The LC-MS/MS method for the determination of the galactolipids (MGDG, DGDG) was validated in the absence of matrices (base calibration). Validation for MGDG was performed using the analyte *m*/*z* 764 (for DGDG *m*/*z* 954) as representative of all MGDG and DGDG. Linearity, accuracy, and precision of the method was determined by analysis of a 10-point calibration curve. This process was repeated over four different days (*n*  =  4). The limit of detection (LOD) was determined by the calibration method. A calibration curve (*n*  =  4) in the range of the presumed detection limit was established and measured (*n*  =  3). The parameters of the resulting regression line were then used to determine LOD and limit of quantitation (LOQ).

### 4.9. Determination of Antioxidant Capacity

For the analysis of the antioxidant activity, samples were prepared with a two-step extraction. The extraction procedure was developed based on a method described by Li et al. [72] and was used with the following modifications: approximately 500 mg freeze-dried powder was weighed into a tube containing 10 mL enzyme solution (0.4 mg lysozyme· mL^−1^, Sørensen buffer; pH 7.4) and mixed for 45 min at 37 °C. The tube was centrifuged at 3226× *g* for 15 min at 15 °C. The residue was re-suspended with 80% ethanol (*v*/*v*), stirred for 1 min, and centrifuged under the same conditions as previously mentioned. Both supernatants were analyzed for total phenolic content (TPC), a well-known indicator of antioxidant activity, and Trolox equivalent antioxidant capacity (TEAC). The total amount was calculated as the sum of the two extracts.

### 4.10. Statistical Analysis

Statistical analysis was performed with OriginPro 2021 (OriginLab Corp., Northampton, MA, USA). Differences among the application levels were evaluated using two-way ANOVA test, and the post-hoc Scheffe test was selected to evaluate probable differences among groups. A level of *p* < 0.05 was considered significant. The results were presented as means and the standard errors of means.

## 5. Conclusions

It is well-known that membrane lipids are affected by abiotic stress factors. S deficiency adversely affects the overall growth and development of lettuce plants. In this study, an influence on the composition of sulfolipids and galactolipids, present especially in the thylakoid membrane, was shown. During long-term S deprivation, the green and red lettuce produced a vast number of individual lipids with a low degree of unsaturated fatty acids such as SQDG-765 and SQDG-793 with at least one saturated fatty acid (14:0 or 16:0) or, e.g., palmitoleic acid (16:1) or oleic acid (18:1) bound. Moreover, higher ratios of individual LOH were detected under similar stress conditions. Consequently, they might act as markers of sulfur-related stress or even other stress factors. To the best of our knowledge, this is the first time LOH has been determined when attached to selected SQDG.

## Figures and Tables

**Figure 9:**
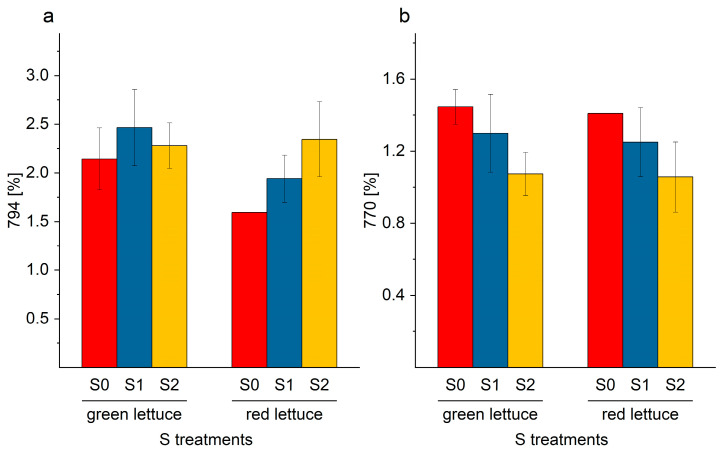
(**a**,**b**). Content (%) of individual monogalactosyl diacylglycerol derivates (**a**) MGDG-794; and (**b**) MGDG-770, in green and red multi-leaf lettuce grown under different S treatments. Values represent the mean ± SD of independent replicates of lettuce treated with S0: 0 mM (*n*_green leaf_ = 4; *n*_red leaf_ = 1), S1: 1 mM (*n*_green leaf_ = 6; *n*_red leaf_ = 5), and S2: 1.5 mM (*n*_green leaf_ = 5; *n*_red leaf_ = 2) K_2_SO_4_, respectively.

**Table 1:** MRM transitions and MS parameters in positive ion mode for MGDG. DT: dwell time; DP: de-clustering potential; EP: entrance potential; CE: collision energy; FA: proposed fatty acid composition.

Analyte [M+ NH_4_^+^]^+^	RT (min)	MRM (*m*/*z*)	DT	DP (V)	EP (V)	CE (V)	FA
742	25.9	742.5 → 307.5	200	86	10	37	16:0/16:3
		742.5 → 313.5	200	86	10	37	
		742.5 → 563.5	200	86	10	17	
764	16.1	764.5 → 335.5	200	56	10	43	16:3/18:3
		764.5 → 567.5	200	56	10	27	
		764.5 → 585.5	200	56	10	25	
766	19.7	766.5 → 335.5	200	76	10	35	16:2/18:3
		766.5 → 569.5	200	76	10	33	
		766.5 → 587.5	200	76	10	23	
768	25.5	768.5 → 309.5	200	76	10	47	16:1/18:3
		768.5 → 571.5	200	76	10	27	
		768.5 → 589.5	200	76	10	37	
770	36.9	770.5 → 261.5	200	71	10	51	16:0/18:3
		770.5 → 313.5	200	71	10	49	
		770.5 → 573.5	200	71	10	52	
792	20.8	792.5 → 261.5	200	51	10	45	18:3/18:3
		792.5 → 335.5	200	51	10	45	
		792.5 → 613.5	200	51	10	27	
794	26.8	794.5 → 337.5	200	81	10	43	18:2/18:3
		794.5 → 335.5	200	81	10	47	
		794.5 → 597.5	200	81	10	25	
796	30.2	796.5 → 337.5	200	56	10	55	18:2/18:2
		796.5 → 537.5	200	56	10	37	
		796.5 → 613.5	200	56	10	52	

**Table 2:** MRM transitions and MS parameters in positive ion mode for DGDG. DT: dwell time; DP: declustering potential; EP: entrance potential; CE: collision energy; FA: proposed fatty acid composition.

Analyte [M+ NH_4_^+^]^+^	RT (min)	MRM (*m/z*)	DT	DP (V)	EP (V)	CE (V)	FA
904	17.6	904.6 → 233.0	200	121	10	43	16:3/16:0
		904.6 → 563.4	200	121	10	35	
		904.6 → 545.6	200	121	10	37	
926	12.2	926.6 → 233.0	200	126	10	41	16:3/18:3
		926.6 → 261.2	200	126	10	57	
		926.6 → 585.0	200	126	10	31	
928	14.3	928.6 → 587.6	200	141	10	29	16:3/18:2
		928.6 → 233.2	200	141	10	37	
		928.6 → 263.2	200	141	10	57	
932	23.8	932.6 → 573.4	200	121	10	31	16:0/18:3
		932.6 → 591.2	200	121	10	27	
		932.6 → 313.0	200	121	10	41	
934	31.7	934.6 → 263.1	200	116	10	43	16:1/18:1
		934.6 → 575.2	200	116	10	41	
		934.6 → 593.6	200	116	10	25	
904	17.6	904.6 → 233.0	200	121	10	43	16:3/16:0
		904.6 → 563.4	200	121	10	35	
		904.6 → 545.6	200	121	10	37	
926	12.2	926.6 → 233.0	200	126	10	41	16:3/18:3
		926.6 → 261.2	200	126	10	57	
		926.6 → 585.0	200	126	10	31	
928	14.3	928.6 → 587.6	200	141	10	29	16:3/18:2
		928.6 → 233.2	200	141	10	37	
		928.6 → 263.2	200	141	10	57	
932	23.8	932.6 → 573.4	200	121	10	31	16:0/18:3
		932.6 → 591.2	200	121	10	27	
		932.6 → 313.0	200	121	10	41	
934	31.7	934.6 → 263.1	200	116	10	43	16:1/18:1
		934.6 → 575.2	200	116	10	41	
		934.6 → 593.6	200	116	10	25	
954	15.0	954.6 → 333.0	200	56	10	39	18:3/18:3
		954.6 → 613.2	200	56	10	55	
		954.6 → 596.6	200	56	10	35	
956	18.4	956.6 → 615.4	200	136	10	27	18:2/18:3
		956.6 → 335.2	200	136	10	55	
		956.6 → 595.4	200	136	10	37	
958	23.5	958.6 → 261.4	200	136	10	46	18:1/18:3
		958.6 → 617.2	200	136	10	27	
		958.6 → 337.4	200	136	10	52	

**Table 3:** Contents of individual SQDG (%) in relation to total SQDG contents in green and red multi-leaf lettuce plants grown under different S treatments, respectively. Values represent the mean ± SD of independent replicates of lettuce treated with S0: 0 mM, S1: 1 mM, and S2: 1.5 mM K_2_SO_4_, respectively. Different letters suggest significant differences (*p* ≤ 0.05). Abbreviations SQDG: sulfoquinovosyl diacylglycerol derivatives, *m*/*z*: mass to charge ratio.

Lettuce Species	Individual SQDG *m*/*z*	SQDG Content at Varied S Treatments [mM]			Enrichment Factor (C_1_._5mM_/c_0mM_)
		S0 [%] ^1^	S1 [%] ^2^	S2 [%] ^3^	
Red multi-leaf	765	0.6 ^a^	0.2 ± 0.01 ^b^	0.3 ± 0.00 ^c^	3.3
	787	0.2 ^a^	0.4 ± 0.03 ^b^	0.3 ± 0.02 ^ab^	10.6
	789	0.3	0.4 ± 0.02	0.4 ± 0.04	8.6
	791	0.7	0.7 ± 0.06	0.8 ± 0.11	8.2
	793	14.1 ^a^	8.1± 1.06 ^b^	8.5 ± 1.40 ^b^	4.3
	801	0.2	0.2 ±0.01	0.2 ± 0.00	8.2
	803	0.1 ^a^	0.1 ± 0.01 ^b^	0.1 ± 0.01 ^ac^	7.5
	813	1.5 ^a^	2.8 ± 0.26 ^b^	2.9 ± 0.49 ^b^	14.0
	815	31.1	39.1 ± 3.51	41.6 ± 5.05	9.7
	817	1.8 ^a^	1.6 ± 0.16 ^ab^	2.5 ± 0.02 ^c^	9.6
	819	0.5 ^a^	0.9 ± 0.11 ^ab^	1.1 ± 0.14 ^b^	16.2
	821	0.4	0.5 ± 0.07	0.6 ± 0.08	9.3
	837	8.1 ^a^	15.6 ± 1.64 ^b^	12.7 ± 0.43 ^ab^	11.3
	839	4.4	3.7 ± 0.59	4.1 ± 0.37	6.6
	841	3.0	2.8 ± 0.38	3.1 ± 0.14	7.4
	843	2.9 ^ab^	2.4 ± 0.20 ^a^	3.4 ± 0.33 ^b^	8.4
	845	0.4 ^ab^	0.5 ± 0.08 ^a^	0.7 ± 0.10 ^b^	12.1
	867	0.2	0.1 ± 0.02	0.1 ± 0.02	3.9
	555	6.6	6.2 ± 1.25	3.5 ± 0.65	3.8
	831	19.2 ^a^	11.1 ± 1.53 ^b^	10.8 ± 1.67 ^b^	4.1
	833	1.3 ^a^	0.6 ± 0.07 ^b^	0.7 ± 0.19 ^b^	3.7
	853	2.1	2.3 ± 0.28	1.8 ± 0.16	6.0
	831	15.6 ± 0.83	13.3 ± 1.28	10.0 ± 1.73	4.8
Green multi-leaf	833	1.3 ± 0.24	0.9 ± 0.09	0.5 ± 0.15	5.0
	853	2.4 ± 0.68	2.7 ± 0.28	2.1 ± 0.37	3.0

^1^ Results of one biological plant, ^2^ Results of three different biological replicates, ^3^ Results of two different biological replicates.

**Table 4:** Contents of individual monogalactosyl diacylglycerol (MGDG) and digalactosyl diacylglycerol (DGDG) (%) in relation to total MGDG or DGDG contents in green and red multi-leaf lettuce plants grown under different S treatments, respectively. Values represent the mean ± SD of independent replicates of lettuce treated with S0: 0 mM, S1: 1 mM, and S2: 1.5 mM K_2_SO_4_, respectively. Different letters suggest significant differences (*p* ≤ 0.05). Abbreviations MGDG: monogalactosyl diacyglycerol derivatives, DGDG: digalactosyl diacylglycerol derivatives, m/z: mass to charge ratio.

Individual MGDG or DGDG *m*/*z*	Green Lettuce			Red Lettuce		
	Galactolipid Content at Varied S Treatments [mM]			Galactolipid Content at Varied S Treatments [mM]		
	S0 ^1^	S1 ^2^	S2 ^3^	S0 ^4^	S1 ^5^	S2 ^6^
MGDG						
764 [%]	0.11 ± 0.0	0.14 ± 0.0	0.15 ± 0.0	0.13	0.16 ± 0.0	0.19 ± 0.0
766 [%]	0.30 ± 0.0	0.28 ± 0.0	0.26 ± 0.0	0.24	0.25 ± 0.0	0.30 ± 0.1
768 [%]	0.41 ± 0.1	0.45 ± 0.1	0.39 ±0.0	0.38	0.39 ± 0.0	0.38 ±0.0
770 [%]	1.45 ± 0.1	1.30 ±0.2	1.07 ± 0.1	1.41	1.25 ± 0.2	1.06 ± 0.2
792 [%]	95.04 ± 0.4	94.77 ± 0.7	95.38 + 0.4	95.84	95.60 ± 0.5	95.41 ± 0.7
794 [%]	2.14 ± 0.3	2.47 ± 0.4	2.28 ± 0.2	1.60	1.94 ± 0.2	2.35 ± 0.4
796 [%]	0.56 ± 0.1	0.60 ± 0.1	0.47 ± 0.1	0.41	0.42 ± 0.1	0.32 ± 0.0
DGDG						
926 [%]	0.07 ± 0.0	0.09 ± 0.0	0.10 ± 0.0	0.07	0.12± 0.0	0.101± 0.0
928 [%]	0.12 ± 0.0	0.13 ± 0.0	0.16 ± 0.0	0.12	0.15 ± 0.0	0.17 ± 0.0
932 [%]	26.50 ± 1.7	24.98 ± 2.4	24.96 ± 0.6	30.25	28.43 ± 1.2	30.67 ± 2.0
934 [%]	10.67 ± 1.7 ^a^	9.38 ± 0.8 ^a^	7.3 ± 0.3 ^b^	12.13 ^a^	7.86 ± 0.7 ^b^	4.97 ± 0.2 ^c^
954 [%]	51.31 ± 2.1	52.14 ± 1.6	54.97 ± 1.6	48.71	52.77 ± 0.9	54.10 ± 4.3
956 [%]	5.62 ± 0.7	6.38 ± 0.5	6.13 ± 0.8	4.48	5.82 ± 0.6	6.45 ± 1.5
958 [%]	5.70 ± 0.6	6.90 ± 0.4	6.44 ± 0.4	4.24	4.86 ± 0.14	3.53 ± 0.6

^1^ Results of four biological replicates, ^2^ Results of six biological replicates, ^3^ Results of five biological replicates, ^4^ Results of one biological plant, ^5^ Results of three different biological replicates, ^6^ Results of two different biological replicates.

**Table 5:** Evaluation of the total phenolic contents (TPC) and the antioxidant capacity, determined by Trolox equivalents antioxidant capacity (TEAC) content in green and red multi-leaf lettuce plants grown under different S treatments, respectively. Values represent the mean ± SD of independent replicates of lettuce treated with S0: 0 mM, S1: 1 mM, and S2: 1.5 mM K_2_SO_4_, respectively. Different letters suggest significant differences (*p* ≤ 0.05).

Parameter	Green Lettuce			Red Lettuce		
	S0 ^1^	S1 ^2^	S2 ^3^	S0 ^4^	S1 ^5^	S2 ^6^
TPC [mg GAE g^−1^ DM]	1.11 ± 0.3	0.75 ± 0.06	0.71 ± 0.1	2.2	2.0 ± 0.4	1.5
TEAC [mM TE g^−1^ DM]	2.14 ± 0.44	1.55 ± 0.2	1.37 ± 0.1	5.18 ^a^	3.73 ± 0.5 ^b^	3.88 ^a,b^

^1^ Results of four biological replicates, ^2^ Results of six biological replicates, ^3^ Results of five biological replicates, ^4^ Results of one biological plant, ^5^ Results of five different biological replicates, ^6^ Results of one biological plant.

## Data Availability

The datasets presented in this study are available upon request from the corresponding author.
